# Clinical study of free folded fibular flap reconstruction combined with implant placement for restoration of mandibular defects and occlusal relationships

**DOI:** 10.3389/fonc.2026.1909133

**Published:** 2026-08-28

**Authors:** Zhenghao Ma, Zhiqiang Pan, Yunqi Chen, Luwen Song, Lina Jiang, Jiancheng Li

**Affiliations:** 1 Department of Oral and Maxillofacial Surgery, The First Affiliated Hospital of Bengbu Medical University, Bengbu, Anhui, China; 2 School of Stomatology, Bengbu Medical University, Bengbu, Anhui, China

**Keywords:** folded fibula flap, implant restoration, mandibular reconstruction, occlusal rehabilitation, stereophotogrammetry

## Abstract

**Objective:**

To investigate the clinical efficacy of using a digitally designed vascularized folded fibula combined with implant placement, and applying stereophotogrammetry for impression taking to reconstruct mandibular defects and restore patients’ occlusal relationships.

**Methods:**

The clinical data of 24 patients treated between January 2020 and February 2024 were analyzed, including 16 cases of benign mandibular tumors, 4 cases of medication-related osteonecrosis of the jaw (MRONJ), and 4 cases of osteoradionecrosis (ORN). Three-dimensional reconstruction of the mandible and fibula was performed with digital technology assistance to simulate mandibular tumor resection, fibular harvesting, and implant placement. Various surgical guides were designed and used to complete mandibular reconstruction and implant placement. Phase II implant surgery was performed 4–6 months postoperatively, and stereophotogrammetry was used to obtain impressions for digital design and fabrication of implant crowns to complete the implant-supported prosthetic restoration. Follow-up visits were conducted at 6 months, 1 year, and 2 years after delivery of the implant-supported prosthesis.

**Results:**

Regarding mandibular defect types, there were 13 cases of Brown Class I defects, 5 cases of Brown Class II defects, and 6 cases of Brown Class III defects. A total of 78 implants were placed in the 24 patients. Six months after delivery of the implant-supported prostheses, all implants and prostheses remained intact. At the 1-year follow-up, one patient presented with a loose implant crown due to loosening of the fixation screw. At the 2-year follow-up, one patient showed significant implant mobility, and the implant was removed.

**Conclusion:**

For patients with segmental mandibular defects caused by benign lesions and jaw osteonecrosis, the use of a digitally designed vascularized folded fibula combined with implant placement to reconstruct mandibular defects, together with stereophotogrammetry for impression taking, can effectively reconstruct mandibular morphology and occlusal relationships. For functional reconstruction of non-neoplastic mandibular defects or after benign tumor resection, this technique is a precise and reliable treatment option.

**Trial registration:**

The study was registered in the Chinese Clinical Trial Registry database on August 21, 2020, under the registration number ChiCTR2000035932.

## 1 Introduction

Mandibular defects are commonly seen following tumor resection, osteonecrosis of the jaw, and trauma. They are often accompanied by tooth loss, leading to severe functional impairments in chewing, swallowing, and opening and closing the mouth, as well as facial collapse and scarring deformities, which significantly reduce patients’ quality of life (1, 2). In the reconstruction of mandibular defects, the restoration of proper occlusal function has become a key quality-of-life measure for patients, placing extremely high demands on the restoration of occlusal function (3, 4). In the past, oral surgeons often used a single-layer free fibula graft to reconstruct mandibular defects; however, its insufficient height was not conducive to implant-supported restoration. To achieve ideal occlusal reconstruction, with the recent advancements in microsurgery and digital technology, the vascularized folded fibula graft has become the mainstream method for reconstructing segmental mandibular defects due to its good moldability and ability to support implants simultaneously (5–7). Restoring occlusal function requires not only precise design and placement of the fibula but also the acquisition of accurate impressions in the complex postoperative oral environment (8, 9). Stereophotogrammetry utilizes a stereoscopic camera to capture multiple target points on a stereophotogrammetric rod, calculating the virtual coordinates and orientations of multiple abutments. This method efficiently determines the relative positions of multiple abutments and facilitates the digital design of implant crowns, thereby enabling more precise reconstruction of mandibular morphology and occlusal function (10–12). In this study, digital technology was used to assist in reconstructing the patient’s mandibular defect with a vascularized folded fibula graft and simultaneous implant placement. Postoperatively, stereophotogrammetry was employed to take impressions for the design of implant crowns, thereby restoring the patient’s occlusal relationship and achieving satisfactory clinical outcomes, as reported below.

## 2 Materials and methods

### 2.1 General information

Twenty-four patients who required mandibular lesion resection and were scheduled for occlusal reconstruction at the Department of Oral and Maxillofacial Surgery of our hospital between January 2020 and February 2024 were enrolled. Among them, 16 patients had benign mandibular tumors, 4 had medication-related osteonecrosis of the jaw (MRONJ), and 4 had osteoradionecrosis (ORN). The study was approved by the Ethics Committee of Bengbu Medical University, and written informed consent was obtained from all patients.

### 2.2 Surgical method

#### 2.2.1 Preoperative preparation

1. Imaging studies: Preoperative CT angiography (CTA) was performed to assess the anatomical structure of the fibular artery and its perforating branches, and the lower leg with superior perforating vessels was selected as the donor site. Color Doppler ultrasound (CDU) was used to identify the origin of the dominant perforating vessels from the fibular artery, thereby determining the center of the fibular osteotomy and the subcutaneous course of the cutaneous perforators. Infrared thermography (IRT) was employed to identify the subcutaneous exit points of the cutaneous perforators and their blood supply range.
2. Surgical guide preparation: CT data were imported in DICOM format into virtual surgery software. The extent of the mandibular lesion was marked on a case-by-case basis on the three-dimensional reconstructed model: for benign tumors, the osteotomy line was set 5 mm beyond the tumor margin; for MRONJ or ORN, the necrotic bone range was marked and the osteotomy line was placed in healthy bone. Following virtual resection, the implant placement positions were designed according to the Brown classification of mandibular defects (13): for Brown Class I defects, 2 implants were placed, corresponding to the mandibular first molar and premolars; for Brown Class II defects, 4 implants were placed, corresponding to the mandibular first molar, premolars, canine, and lower anterior teeth; for Brown Class III defects, 4–6 implants were placed to restore the mandibular dentition defect. Simultaneously, when designing the fibular osteotomy lines, the distal osteotomy was performed at least 6–8 cm proximal to the lateral malleolus, with a minimum preserved distance of approximately 5 cm from the distal tibiofibular syndesmosis, to maintain ankle joint stability and distal tibiofibular ligament attachment. Based on the above planning, mandibular osteotomy guides, fibula shaping guides, and other surgical guides were designed; 3D printing technology was used to print the mandibular model and guides; and reconstruction titanium plates and micro-titanium plates were pre-bent on the mandibular model.

#### 2.2.2 Mandibular reconstruction

All surgical procedures were performed by a combined Oral and Maxillofacial Surgery–Microsurgery team: mandibular lesion resection and implant placement were performed by oral and maxillofacial surgeons, while fibula flap harvest and microvascular anastomosis were performed by microsurgery-qualified surgeons under a surgical microscope.

1. An incision was made at the normal mucosa of the buccal/labial vestibular sulcus of the mandible, the periosteum was dissected, and the muscular attachments of the mandible were severed. The surgical guide was positioned according to the preoperative design, the mandible was resected, and the feeding artery and draining vein were preserved simultaneously.
2. After exposing the fibula, the skin paddle was harvested using a fasciocutaneous technique: the skin, subcutaneous tissue, and superficial layer of the deep fascia were incised along the designed line; the skin, subcutaneous fat, and deep fascia of the lower leg were elevated as a single unit from the surface of the fibular intermuscular septum, supplied by the fibular artery musculocutaneous perforators, with at least 2–3 cm of fascial tissue preserved around the perforating vessels. Subsequently, the periosteum was incised longitudinally along the medial border of the fibula, preserving the integrity of the lateral periosteum, peroneus longus, and soleus muscular cuff. At each planned osteotomy site (folding, proximal, and distal), a subperiosteal osteotomy was performed, with a limited periosteal incision followed by careful elevation of the periosteum using a raspatory, thereby protecting the periosteal blood supply on both sides of the osteotomy; the lateral periosteal-muscular cuff was left intact as an undisturbed soft-tissue hinge. A full-thickness cortical osteotomy was first performed at the predetermined folding site but not completely detached, maintaining the shared blood supply to both bone segments; osteotomies were then completed at the proximal and distal osteotomy lines (14).
3. The harvested fibula was shaped with the aid of the shaping guide. The periosteum and muscle on the contact surfaces of the two bone segments were stripped, while the periosteal-muscular cuff was preserved around the remaining circumference, forming a folded fibular graft. The lower bone segment was used to restore mandibular continuity and facial contour, while the upper bone segment was used to reconstruct the alveolar bone contour and height. Finally, the graft was secured with titanium plates.
4. Vascular anastomosis: The vascular pedicle of the fibula flap in this series consisted of the peroneal artery and its two venae comitantes. The peroneal artery was dissected and freed intraoperatively to the bifurcation of the posterior tibial artery, with the vascular pedicle length preserved at approximately 6–8 cm, ensuring no torsion or tension. The facial artery was the first choice for the recipient artery, with the lingual artery as the alternative; the anterior facial vein was the first choice for the recipient vein, followed by the external jugular vein. All anastomoses were performed under a surgical microscope using 9–0 or 10–0 nylon sutures in an end-to-end fashion. After arterial anastomosis was completed, the vein was opened first, followed by the artery, and the color of the skin paddle, capillary refill, and bleeding were observed.
5. Donor-site management: After fibular harvest, the residual bone stump was sealed with bone wax for hemostasis. The peroneus longus, soleus, and lower leg fascia were carefully sutured, a negative-pressure drain was placed subcutaneously, and the skin incision was closed in layers. Postoperatively, the donor lower leg was compressed with an elastic bandage, and the affected limb was elevated.

#### 2.2.3 Implant placement

The Straumann implant system (4.1 × 10 mm, BLT) was selected. According to the preoperative design, the following steps were performed at the designated implant positions: marking, reaming, shaping, tapping, implant insertion, and screw coverage. The implant was then submerged, and the wound was sutured.

#### 2.2.4 Stage II implant surgery

Four to six months after implant placement, an incision was made at the healing site between the skin paddle and the mucosa under local anesthesia. The skin paddle was reflected toward the lingual side, subcutaneous fat tissue was removed, the skin margin was trimmed, and the buccal periosteum was undermined and dissected. The cover screw was removed, the healing abutment was placed, and the buccal periosteum was sutured to the skin margin. Titanium fixation plates were removed in some patients when clinically indicated.

### 2.3 Impression taking and digital crown fabrication

The healing abutments were removed, and iCamRefs were attached to the implants. The SHINING 3D intraoral scanner was then used to scan the patient’s mouth and capture the soft tissue contour data of the gingiva and the transplanted flap. The iCamRefs were removed and iCamBodies scan bodies were attached. After calibrating the scanning system, the handheld stereophotogrammetry scanner was held at a distance of 15–30 cm and moved slowly from one side of the edentulous segment to the other to capture the iCamPosition data (which includes the three-dimensional position and orientation information of the implants). The iCamPosition data were matched with the intraoral soft tissue data: in the dedicated image scanning software iScan3D Dental, the iCamPosition spatial data were imported into the soft tissue coordinate system acquired with iCamRefs, and the data matching was completed to finalize the digital impression. Subsequently, a titanium framework was designed in the software and fabricated using CAD/CAM technology. After verifying a proper passive fit with the framework try-in, the crowns were designed on the framework according to the opposing dentition.

### 2.4 Postoperative follow-up

The follow-up protocol included: ① Flap survival assessment: observation of skin paddle color, texture, and elasticity; ② Donor-site complications: observation of postoperative wound infection, dehiscence, peroneal nerve injury, and other relevant complications; ③ Implant evaluation: at 6 months, 1 year, and 2 years after delivery of the implant-supported prosthesis, implant status was assessed according to Albrektsson’s success criteria (no clinical mobility, no persistent pain or infection, no radiolucency on radiographs, vertical bone loss < 1.5 mm during the first year and < 0.2 mm annually thereafter), and marginal bone loss (MBL) was measured; ④ Prosthetic evaluation: mechanical and biological complications were recorded at 6 months, 1 year, and 2 years postoperatively.

## 3 Results

In this study, following mandibulectomy, 13 patients had Brown Class I defects, 5 had Class II defects, and 6 had Class III defects.

Flap survival and bone healing: All 24 free fibula flaps survived, with a flap survival rate of 100%. Postoperative imaging at 6 months showed that 22 patients achieved bony union between the folded fibula and the mandibular stump as well as between the upper and lower fibular segments, with no nonunion or fibrous union observed. In 2 patients, unilateral bony union was achieved with contralateral fibrous union; clinically, no segmental mobility was observed. All 24 donor-site lower leg incisions healed primarily, with no wound infection, dehiscence, or hematoma formation. In the early postoperative period, one patient developed mild swelling of the donor lower leg, which resolved within one week after limb elevation and compression bandaging. All patients exhibited normal dorsiflexion and eversion of the foot postoperatively, with no foot drop or sensory deficits over the lateral lower leg or dorsum of the foot suggestive of common peroneal nerve injury.

Implant outcomes: A total of 78 implants were placed in the 24 patients. Six months after delivery of the implant-supported prostheses, no implant mobility or radiolucency was observed, and no patient reported pain or paresthesia around the implants. At the 1-year follow-up, one patient presented with loosening of an implant crown; examination revealed a loose retention screw, which was removed and retightened, resolving the loosening. The mean marginal bone loss (MBL) was 0.77 ± 0.32 mm. No peri-implantitis was observed. At the 2-year follow-up, a radiolucency was detected around the implant on a panoramic radiograph in one patient. After removal of the implant-supported prosthesis, significant implant mobility was observed, and the implant was subsequently extracted. The mean MBL was 1.12 ± 0.45 mm.

Complications: Mechanical complications occurred in one case (screw loosening at 1 year); biological complications occurred in one case (peri-implantitis leading to bone resorption and implant mobility at 2 years)(**Table 1**).

**Table 1:** Demographic, pathological, surgical, and outcome characteristics of the patients (n = 24).

Variable	Value/description
Total patients, n	24
Age, mean ± SD (range), years	47.5 ± 15.9 (27–74)
Sex, n (%)	
Male	11 (45.8)
Female	13 (54.2)
Pathology, n (%)	
Benign mandibular tumor	16 (66.7)
Ameloblastoma	11 (45.8)
Ossifying fibroma	3 (12.5)
Odontogenic myxoma	1 (4.2)
Giant cell tumor	1 (4.2)
MRONJ	4 (16.7)
ORN	4 (16.7)
Mandibular defect (Brown classification), n (%)	
Class I	13 (54.2)
Class II	5 (20.8)
Class III	6 (25.0)
Total implants placed, n	78
Implants per patient, mean ± SD (range)	3.3 ± 1.5 (2–6)
Implant system	Straumann BLT (4.1 × 10 mm)
Recipient artery for anastomosis, n (%)	
Facial artery	23 (95.8)
Lingual artery	1 (4.2)
Recipient vein for anastomosis, n (%)	
Anterior facial vein	23 (95.8)
Common facial vein	1 (4.2)
Flap survival, n (%)	24 (100)
Bone healing at 6 months, n (%)	
Bilateral bony union	22 (91.7)
Unilateral bony union with contralateral fibrous union	2 (8.3)
Peroneal nerve injury, n (%)	0 (0)
Implant/prosthetic complications	
Screw loosening	1 (4.2)
Peri-implantitis with implant loss	1 (4.2)
Marginal bone loss (MBL), mean ± SD, mm	
At the 1-year follow-up	0.84 ± 0.32
At the 2-year follow-up	1.05 ± 0.45

## 4 Representative case

A 28-year-old male presented with progressive swelling of the right mandible for over six months. An incisional biopsy of the mandibular mass was performed after admission, and the pathological diagnosis was ameloblastoma of the mandible with infection and abundant inflammatory cell infiltration.

Preoperative infection management: Aerobic and anaerobic cultures were obtained from wound secretions, and no bacterial growth was observed after 72 hours of incubation. Considering that ameloblastoma was accompanied by local inflammation and abundant inflammatory cell infiltration, and that the common oral and maxillofacial flora consists of Gram-positive cocci and anaerobic bacteria, empirical antibiotic therapy was administered: intravenous amoxicillin-clavulanate combined with metronidazole. Surgery was performed after local redness and swelling subsided and white blood cell (WBC) count and C-reactive protein (CRP) levels returned to normal.

Surgical procedure: Digital intraoral approach mandibular tumor resection and free fibula flap mandibular reconstruction were performed. The postoperative incision healed primarily, with no signs of common peroneal nerve injury. The mandibular defect type was Brown Class III. Straumann BLT implants (4.1 × 10 mm) were placed, totaling 5 implants, with intraoperative primary stability torque values ≥ 35 N·cm. At the 6-month follow-up after implant placement, the patient met the infection control criteria for second-stage surgery, and Stage II implant surgery was subsequently performed.

Impression and restoration: Two weeks after Stage II surgery, an impression was obtained using stereophotogrammetry. After matching the iCamPosition with soft tissue data, a titanium framework and resin crowns were designed and fabricated using CAD/CAM technology. Following delivery of the implant-supported prosthesis, the occlusal relationship was favorable, and passive fit was confirmed.

**Figure 1:**
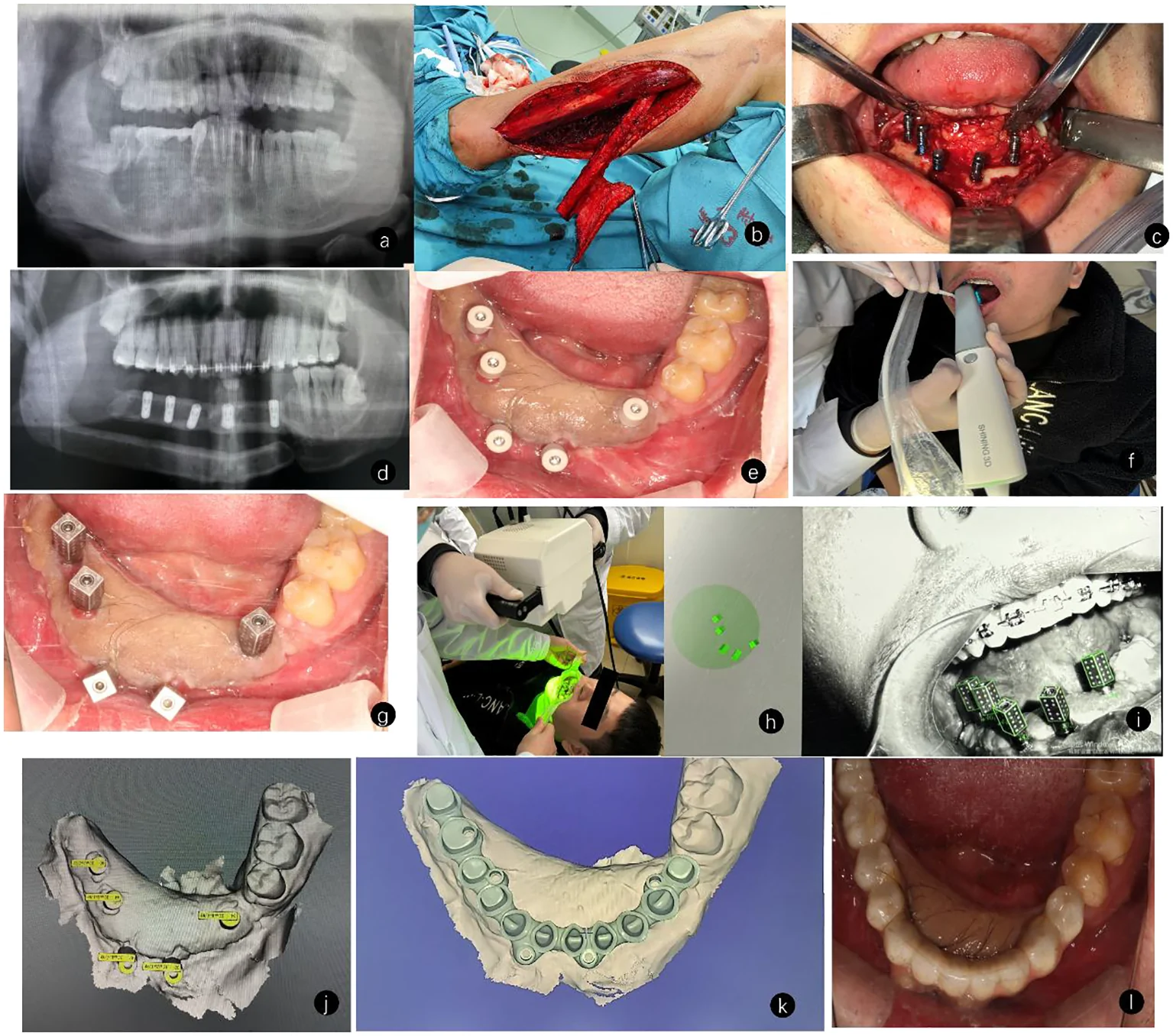
Representative case **(A)** Panoramic radiograph **(B)** Intraoperative harvesting of a fibula flap for mandibular reconstruction. **(C, D)** Implant placement and the corresponding panoramic radiograph. **(E–I)** Acquisition of digital data using SPG combined with IOS. **(J)** Matching iCamPosition spatial data with soft tissue intraoral scan data. **(K)** Digital design of implant prosthesis. **(L)** Intraoral view after implant denture placement.

Follow-up: At the 6-month follow-up after delivery of the implant-supported prosthesis, and the patient was satisfied with the restoration of facial contour and masticatory function, with normal speech clarity. At the 1-year and 2-year follow-ups, the MBL was 0.63 mm and 0.76 mm, respectively; the implants met Albrektsson’s success criteria, and no mechanical or biological complications were observed (**Figure 1**).

## 5 Discussion

Thanks to its reliable bone healing capacity and good plasticity, the vascularized free fibular osteocutaneous flap has become the mainstream method for repairing segmental mandibular defects (15, 16). However, there remains a significant gap between restoring the bony framework and achieving fully functional occlusal reconstruction. The precise restoration of occlusal function depends not only on whether the bone volume, morphology, and spatial position of the reconstructed fibula can provide an ideal bony foundation for the implant but also heavily relies on the ability to obtain digital impressions during the prosthetic phase that faithfully reproduce the three-dimensional orientation of the intraoral implant, thereby enabling the fabrication of implant restorations with a high degree of passive fit. To address this, we have organically integrated the digital folded fibula technique with stereophotogrammetry to explore the establishment of a precise occlusal reconstruction protocol covering the entire workflow from surgical reconstruction to prosthetic fabrication.

Following mandibular tumor resection, due to the loss of attachment of the buccal and lingual muscle groups and soft tissue collapse, single-layer fibula reconstruction often suffers from defects such as insufficient height and weak support for intraoral soft tissues, making it difficult to meet the bone volume requirements for subsequent implant-supported restorations. Furthermore, the limited space above a single-layer fibular graft often results in an unfavorable crown-to-root ratio after implant placement, increasing the risk of long-term mechanical complications. In contrast, a double-barrel folded fibular graft can elevate the graft height to a level close to that of the original mandibular alveolar ridge, thereby facilitating the restoration of an appropriate crown-to-root ratio (5–7). However, in long-segment defects involving the mandibular body and ramus—such as Brown Type Ic, IIc, and III—the effective length of the fibular donor segment is inherently limited by the osteotomy range required to avoid compromising distal blood supply and ankle joint stability. Blindly pursuing a full-thickness folded graft may result in insufficient fibular length; simultaneously, the superimposition of two bone segments may exceed the original alveolar ridge height, causing excessive tension on the labial and lingual sides during mucosal suturing, which can impair wound healing and even hinder the establishment of space for subsequent prosthetic restoration.

To address these challenges, we employ a folded fibula design. In this design, the lower fibular segment is positioned along the original inferior border of the mandible, with the restoration of the mandibular contour and facial profile as the baseline; the upper fibular segment does not aim to cover the entire length of the defect but is concentrated in the implant site area of the edentulous region—that is, it covers only the bone segment where the implant is expected to be placed, situated between the implant sites on either side of the defect or between the implant site and the osteotomy end, without extending into the ramus. Under conditions of limited bone harvest length, this approach concentrates the compensatory bone height of the fibula in the critical implant area, thereby meeting the bone height requirements for the implant while avoiding the risk of the folded bone block protruding above the alveolar ridge plane in the anterior region. Furthermore, the distal osteotomy was performed at least 6–8 cm proximal to the lateral malleolus, with a minimum preserved distance of approximately 5 cm from the distal tibiofibular syndesmosis, to maintain ankle joint stability and distal tibiofibular ligament attachment (14, 17). Intraoperatively, prior to aligning and fixing the folded fibula, the periosteum and attached muscles were carefully dissected, and soft tissue in the contact area between the two bone segments was thoroughly removed to ensure parallel alignment and tight contact between the upper and lower fibular segments, thereby promoting primary union between the bone segments. In the 24 cases in this group treated with this protocol, postoperative radiographic examination at 6 months demonstrated that 22 patients achieved bony union between the folded fibula and the mandibular stump as well as between the upper and lower fibular segments, with no nonunion or fibrous union observed; 2 patients achieved unilateral bony union with contralateral fibrous union.

Regarding the selection of the donor site on the lower leg, we relied on findings from CT angiography and color Doppler ultrasound to prioritize the side with superior perforating blood supply and minimal anatomical variation as the donor site. In this group, three patients exhibited no significant difference in the dominance of perforating vessels between the two lower legs. Since the skin paddles in this study were uniformly designed in the upper part of the fibular flap with the vascular pedicle located posterior to the fibular flap, the contralateral lower leg was selected as the donor site in all three cases to facilitate vascular positioning and anastomosis.

The survival of the free fibular osseocutaneous flap depends on precise vascular pedicle anastomosis. In this study, all procedures were performed by a combined oral and maxillofacial surgery–microsurgery team: oral and maxillofacial surgeons performed the mandibular lesion resection and implant placement, while microsurgery-qualified surgeons performed the free fibula flap harvesting and recipient vessel anastomosis under a surgical microscope. The donor site uniformly preserved the peroneal artery and its two venae comitantes to constitute the vascular pedicle, with a length of approximately 6–8 cm. The facial artery was the first choice for the recipient artery, and the anterior facial vein was the first choice for the recipient vein. All anastomoses were performed with 9–0 or 10–0 non-traumatic nylon sutures in an end-to-end fashion. For ORN patients, prior radiation therapy may cause thickening of the facial artery or anterior facial vein wall and luminal stenosis; therefore, preoperative CTA or ultrasound assessment of recipient vessel conditions is required. When necessary, the lingual artery may be selected as an alternative recipient artery, and the external jugular vein or common facial vein as alternative recipient veins. In one of the four ORN patients in this group, anastomosis to the common facial vein was performed due to inadequate outflow through the anterior facial vein, and postoperative flap perfusion remained stable. This suggests that individualized assessment and flexible selection of recipient vessels are critical to ensuring flap survival in complex cases such as osteoradionecrosis. All 24 fibular osseocutaneous flaps in this study survived, with a flap survival rate of 100%.

However, the long-term survival of the skin paddle intraorally remains challenged by histological mismatches: skin lacks the moisture, elasticity, and submucosal glandular secretion of oral mucosa, and prolonged exposure to the oral environment may result in surface hyperkeratosis, color discrepancy, and elastic retraction. In this study, debulking during Stage II implant surgery partially improved the bulkiness of the skin paddle but did not fundamentally alter the histological characteristics of the skin. Literature reports indicate that mucosalization may occur in some cases over the long term, wherein the skin surface is gradually replaced by oral mucosa-like epithelium; however, this process is slow and incomplete. For functionally demanding areas such as the vestibular sulcus or masticatory mucosa, future mucosal grafting may be considered to further improve the restorative outcome (18). Regarding the donor site, all 24 lower leg incisions healed primarily, with no wound infection, dehiscence, or hematoma formation. In the early postoperative period, one patient developed mild swelling of the donor lower leg, which resolved within one week after limb elevation and compression bandaging. All patients exhibited normal dorsiflexion and eversion of the foot postoperatively, with no signs of common peroneal nerve injury, chronic pain, or ankle instability. However, this study did not perform quantitative functional assessment of the donor site (such as ankle range-of-motion measurement or gait analysis), and more objective donor-site outcome indicators should be incorporated in the future.

Occlusal reconstruction is not merely a simple filling of the edentulous area, but rather aims to restore stable and efficient masticatory function as the ultimate goal. Therefore, the determination of the number and location of implants is directly based on the type of defect and the extent of restoration, adhering to function-oriented design principles. The canine region, located at the corner of the dental arch, plays a vital role in supporting the corners of the mouth and maintaining facial symmetry and lip fullness; its aesthetic contribution is self-evident. The first molar, serving as the “occlusal key,” bears the primary masticatory forces, and its position and axis directly determine the functional quality of the entire occlusal reconstruction. However, the oral environment following jaw reconstruction is characterized by the splicing of multiple bone segments, altered anatomical landmarks, and extensive spans of consecutive tooth loss. Relying solely on the surgeon’s visual estimation and experience to determine implant sites is highly prone to errors, leading to unstable occlusal contact in the final restoration and potentially inducing non-axial loading as well as mechanical and biological complications. Therefore, starting from the surgical planning stage, it is essential to utilize virtual surgery and 3D-printed osteotomy guides, positioning guides, and other tools to back-plan the ideal prosthesis position into the virtual implant design, thereby achieving restoration-driven precise implant planning.

Once bone reconstruction and implant placement are complete, another critical challenge arises: how to accurately capture the actual three-dimensional position of the implants as digital data to guide the fabrication of the final prosthesis. Unfortunately, patients who have undergone jaw reconstruction face far more challenging conditions for impression taking than those with conventional dental arch defects: postoperative soft and hard tissue contours are significantly distorted, the vestibular sulcus is shallow or even absent, and the overlying mucosa has become thin and sensitive due to multiple surgeries; mouth opening is often limited by scarring, muscle contractures, or joint issues; and implants typically number 4 to 7, distributed across different regions of the fibula-reconstructed alveolar ridge, with wide spacing between them and varying axial orientations. These factors pose significant challenges to impression accuracy and procedural reproducibility.

Traditional impression techniques rely on elastomeric materials such as alginate or silicone rubber, along with open- or closed-tray systems, to physically replicate the intraoral morphology. While these methods are intuitive and cost-effective, their limitations are drastically magnified under the aforementioned complex conditions: the combination of the tray and impression material readily triggers the patient’s gag reflex, making cooperation difficult; material polymerization shrinkage and minute displacements during the procedure often result in blurred margins at the implant shoulder region, reducing transfer accuracy; the cast plaster models are highly sensitive to impact and fluctuations in temperature and humidity during transport and storage; model breakage or dimensional drift can directly result in poor restoration fit; the technique is highly technique-sensitive, with limited reproducibility among different operators; most critically, physical impressions cannot be directly converted into high-fidelity digital models, making it difficult to seamlessly integrate with fully digital restoration workflows represented by CAD/CAM (19–21).

To overcome this challenge, stereophotogrammetry—a non-contact, instantaneous, and synchronous method for acquiring digital impressions—offers unique advantages. Derived from industrial precision metrology, this technology uses a multi-camera system with at least two lenses to simultaneously capture two-dimensional images of feature points on the target surface from different angles. By applying the principle of parallax, it calculates three-dimensional coordinates to reconstruct a spatially accurate model. When applied to the field of dental implantology, pre-set optical markers (photogrammetric rods) need only be attached to each implant in the mouth. A dedicated handheld or multi-lens fixed camera can then capture the spatial information of all markers in a single session within seconds. Specialized software subsequently calculates the precise position, axis, and depth of each implant.

For the complex and sensitive oral environment following jaw reconstruction, the advantages of stereophotogrammetry are unmatched by traditional techniques: First, the entire process involves no trays or impression materials, completely eliminating stimuli that trigger the gag reflex or cause soft tissue deformation due to pressure. Even patients with limited mouth opening or heightened reflexes can comfortably complete data acquisition; Second, the data acquisition process is highly streamlined. A 3D digital model is generated directly from the intraoral environment, bypassing error-prone physical steps such as material curing, model casting, demolding, and transportation. The actual in-bone orientation of the implants is non-destructively imported into CAD/CAM software with micrometer-level precision, providing a reliable data source for high-precision prosthesis fabrication; Third, the spatial relationships of all implants are simultaneously captured in a single instant, eliminating the need to reconstruct the entire dental arch by stitching together local fields of view—as is required with intraoral scanning. This eliminates cumulative errors that may arise from multi-frame stitching, significantly reducing chairside procedure time compared to traditional methods and markedly decreasing fatigue for both the dentist and the patient; Fourth, the generated files are purely digital, occupying no physical space. They can be instantly stored, remotely transmitted, and losslessly duplicated, without any distortion or loss of information due to environmental temperature and humidity, accidental impact, or the passage of time (22, 23).

Clinical evidence is beginning to support the value of stereophotogrammetry in edentulous implant impressions. An *in vivo* study by Xiao-Jiao Fu et al. (24) confirmed that stereophotogrammetry is significantly more efficient than traditional impression techniques for taking implant impressions. A comparative study on edentulous implant restorations by María Peñarrocha-Diago et al. (25) demonstrated that this technology serves as an effective alternative to traditional methods, with higher satisfaction among both patients and clinicians and a marked reduction in procedure time. The study by Ma et al. (26) further noted that full-arch implant impressions created using stereophotogrammetry demonstrated excellent fidelity and accuracy. Collectively, this evidence indicates that stereophotogrammetry can significantly optimize the impression-taking experience and workflow efficiency in complex implant cases while maintaining or improving accuracy.

The combined application of the digital folded fibula technique and stereophotogrammetry essentially establishes a seamless data link throughout the entire process of “bone reconstruction—implant placement—digital impression—restoration fabrication.” Preoperatively, virtual surgical planning is used to determine the osteotomy, shaping, and fixation protocols for the folded fibula. During surgery, 3D-printed osteotomy guides and positioning guides are utilized to precisely perform folded fibula reconstruction, ensuring adequate bone volume and optimal positioning in the implant site. Following implant placement, stereophotogrammetry is used to capture high-fidelity spatial coordinates of all implants across the dental arch in a single step. This generates a digital impression file that can be directly transmitted to a CAD/CAM system, enabling the virtual design and precise fabrication of the superstructure within the software. The resulting implant-supported fixed prosthesis can be placed in the mouth with exceptional passive fit, achieving a harmonious balance of function and aesthetics.

Study Limitations: This study is a single-center retrospective case series. The enrolled patients were limited to those with benign tumors, MRONJ, or ORN, with relatively conservative tumor resection margins, mild soft tissue defects, and no need for postoperative adjuvant radiochemotherapy. In contrast, mandibular defects following oral and maxillofacial malignant tumor resection are often accompanied by more extensive soft tissue loss, decreased bone tissue blood supply due to postoperative radiotherapy, and impaired healing capacity of peri-implant tissues, with significantly elevated risks of reconstruction and implant restoration complications (16, 23). Therefore, the conclusions of this study cannot be directly extrapolated to malignant tumor patients. Furthermore, this study lacks prospective comparison with traditional approaches such as single-layer vascularized fibula reconstruction or titanium plate reconstruction alone, and it is not yet possible to assert that this technique represents the “ideal” or “standard” protocol for all mandibular defects.

Regarding follow-up and bone healing assessment, the follow-up period in this study was only 2 years, which is insufficient to fully evaluate the long-term survival and functional stability of free fibula grafts. Literature indicates that bone remodeling after vascularized fibula transplantation can continue for more than 5 years, and long-term marginal bone loss and mechanical complications of implants may not become fully apparent until 3–5 years or even longer postoperatively. In this group, two patients presented with unilateral bony union and contralateral fibrous union. Under long-term functional loading, the fibrous union area may progress to delayed union or nonunion, potentially leading to non-axial implant loading, accelerated marginal bone resorption, and even displacement of the grafted bone segment and alteration of the occlusal relationship. Partial synostosis between the folded fibular segments also warrants attention: if complete bony integration between the upper and lower segments is not achieved, axial implant loading may concentrate on a single bone segment, causing stress shielding or intersegmental shear, thereby affecting long-term implant stability. Ishiiet al. (27) noted that the quality of intersegmental healing after double-barrel fibula reconstruction directly influences the implant crown-to-root ratio and the incidence of mechanical complications. Therefore, despite only one implant failure at 2 years, the risk of complications related to poor bone healing in the long term cannot be excluded.

Future Research Directions: Multicenter, prospective randomized controlled studies are needed to clarify the indications and optimal patient populations for this technique in defects of different etiologies. Follow-up should be extended to at least 5 years, and CBCT-based three-dimensional bone healing scores (such as HU value measurement and trabecular orientation analysis) should be introduced to quantitatively assess stump and intersegmental healing quality, rather than relying solely on crude judgments from two-dimensional panoramic radiographs. For malignant tumor patients, the safety and efficacy of this technique must be evaluated separately in the context of postoperative radiochemotherapy.

## 6 Conclusion

In summary, for segmental mandibular defects caused by benign lesions, MRONJ, and ORN, digitally designed vascularized folded fibula combined with implant placement can effectively reconstruct mandibular defects, while stereophotogrammetry solves the challenge of acquiring three-dimensional positional information of implants in complex intraoral environments. The synergy of both techniques achieves digital integration from mandibular reconstruction to implant restoration. Through the folded fibula design, bone height in critical implant areas is concentrated and compensated despite limited donor-site bone volume; with the aid of stereophotogrammetry, the accuracy limitations of traditional impressions under conditions of limited mouth opening and soft tissue deformation are overcome, ultimately yielding acceptable mid-term clinical outcomes. However, this study is a single-center retrospective case series that did not include malignant tumor patients, lacks prospective comparison with single-layer fibula reconstruction, and has a follow-up period of only 2 years. The conclusions are temporarily limited to the aforementioned non-malignant mandibular defect population. Its long-term efficacy and applicability in malignant tumor patients await further validation through multicenter studies with larger sample sizes and longer follow-up periods.

## Data Availability

The datasets generated and analyzed during the current study are not publicly available in online repositories to strictly protect patient privacy and clinical confidentiality. However, anonymized data can be made available from the corresponding author upon reasonable request and subject to further institutional ethical approval.
